# Comparison of the Inhibition of Monoamine Oxidase and Butyrylcholinesterase Activities by Infusions from Green Tea and Some Citrus Peels

**DOI:** 10.1155/2014/586407

**Published:** 2014-08-05

**Authors:** Ayokunle O. Ademosun, Ganiyu Oboh

**Affiliations:** Functional Foods and Nutraceuticals Unit, Department of Biochemistry, Federal University of Technology, PMB 704, Akure 340001, Nigeria

## Abstract

This study sought to investigate the effect of infusions from green tea (*Camellia sinensis*) and some citrus peels [shaddock (*Citrus maxima*), grapefruit (*Citrus paradisi*), and orange (*Citrus sinensis*)] on key enzymes relevant to the management of neurodegenerative conditions [monoamine oxidase (MAO) and butyrylcholinesterase (BChE)]. The total phenol contents and antioxidant activities as typified by their 2,2^′^-azino-bis(3-ethylbenzthiazoline-6-sulphonic acid) (ABTS) radicals scavenging abilities, ferric reducing antioxidant properties, and Fe^2+^ chelating abilities were also investigated. Green tea had the highest total phenol (43.3 mg/g) and total flavonoid (16.4 mg/g) contents, when compared to orange [total phenol (19.6 mg/g), total flavonoid (6.5 mg/g)], shaddock [total phenol (16.3 mg/g), total flavonoid (5.2 mg/g)], and grapefruit [total phenol (17.7 mg/g), total flavonoid (5.9 mg/g)]. Orange (EC_50_ = 1.78 mg/mL) had the highest MAO inhibitory ability, while green tea had the least MAO inhibitory ability (EC_50_ = 2.56 mg/mL). Similarly, green tea had the least BChE inhibitory ability (EC_50_ = 5.43 mg/mL) when compared to the citrus peels' infusions. However, green tea infusions had the strongest highest ABTS radical scavenging ability, reducing power, and Fe^2+^ chelating ability. The inhibition of MAO and BChE activities by the green tea and citrus peels infusions could make them good dietary means for the prevention/management of neurodegenerative conditions.

## 1. Introduction 

Some of the therapeutic targets that have been identified in the management of neurodegenerative conditions include monoamine oxidase (MAO), acetylcholinesterase (AChE), butyrylcholinesterase (BChE), and oxidative stress [1]. The use of MAO inhibitors and their mechanism of action in the management of depression and other neurodegenerative conditions such as Alzheimer's disease (AD) and Parkinson's disease (PD) have been established [2]. Unchecked MAO activity, especially MAO-B, contributes to neurodegeneration leading to Alzheimer's disease development [3]. Furthermore, excessive MAO activity has been linked to increased generation of free radicals in the brain and consequently neuronal damage [3]. Therefore, inhibiting MAO activity is a therapeutic target in the management of AD and other neurodegenerative conditions.

Reduced cholinergic function in the brain has been shown to result in memory impairment in AD [4]. Therefore, the use of AChE and BChE inhibitors to reduce the hydrolysis of acetylcholine is a therapeutic approach in managing neurodegeneration. However, studies have suggested that the joint inhibition of AChE and BChE is preferred to the selective inhibition of AChE in treating AD [5]. More so, the activity of AChE has been shown to decline with AD progression, while BChE activity increases with the progression of the disease [6, 7]. Furthermore, efforts aimed at ameliorating oxidative damage have also been shown to be beneficial in the treatment of neurodegenerative conditions. A combination of cholinesterase inhibition and antioxidative abilities is regarded as an effective therapy in management of such conditions [8]. Green tea and citrus peels have been in use in alternative and complementary medicine as a cheap intervention in the management of some degenerative conditions and they are consumed in form of infusions in many countries.

The inhibition of AChE activity, inhibition of Fe^2+^ induced lipid peroxidation, and radical scavenging abilities of aqueous extracts from some citrus peels had been earlier reported [9]. However, this study sought to compare the effect of infusions from green tea (*Camellia sinensis*) and some citrus peels [shaddock (*Citrus maxima*), grapefruit (*Citrus paradisi*), and orange (*Citrus sinensis*)] on MAO and BChE (key enzymes linked to neurodegenerative conditions) activities, as well as their iron chelating properties* in vitro.*

## 2. Materials and Methods 

### 2.1. Sample Collection

Green tea (*Camellia sinensis*) and some citrus peels shaddock (*Citrus maxima*), grapefruit (*Citrus paradisi*), and orange (*Citrus sinensis*) were collected from the Akure, Southwest, Nigeria. The green tea and peels were sun-dried for 7 days and ground to fine powder.

### 2.2. Infusion Preparation

The infusions were prepared by soaking the ground samples in boiling water for 5 minutes. The resulting mixture was then filtered and the filtrate was stored in the refrigerator for subsequent analysis.

### 2.3. Determination of Total Phenol Content

The total phenol content was determined according to the method of Singleton et al. [10]. Briefly, appropriate dilutions of the extracts were oxidized with 2.5 mL 10% Folin-Ciocalteu's reagent (v/v) and neutralized by 2.0 mL of 7.5% sodium carbonate. The reaction mixture was incubated for 40 minute at 45°C and the absorbance was measured at 765 nm in the spectrophotometer. The total phenol content was subsequently calculated as gallic acid equivalent (GAE).

### 2.4. Determination of Total Flavonoid Content

The total flavonoid content was determined using a slightly modified method reported by Meda et al. [11]; briefly 0.5 mL of appropriately diluted sample was mixed with 0.5 mL methanol, 50 *μ*L 10% AlCl3, 50 *μ*L 1 M potassium acetate, and 1.4 mL water and allowed to incubate at room temperature for 30 min. The absorbance of the reaction mixture was subsequently measured at 415 nm; the total flavonoid content was subsequently calculated.

### 2.5. Enzyme Inhibition Assays 

#### 2.5.1. Monoamine Oxidase (MAO) Inhibition Assay

The assay of MAO and activity measurement of MAO with different concentrations of infusions was performed as per the methods [12, 13], with slight modification. In brief the reaction mixture contained 0.025 M phosphate buffer of pH 7, 0.0125 M semicarbazide, 10 mM benzylamine (pH adjusted to 7), and 0.67 mg of enzyme and appropriate dilutions of the infusions in a total reaction volume of 2 mL. After 30 min, 1 mL of acetic acid was added and boiled for 3 min in boiling water bath followed by centrifugation. The resultant supernatant (1 mL) was mixed with equal volume of 0.05% of 2, 4-DNPH and 2.5 mL of benzene was added after 10 min incubation at room temperature. After separating the benzene layer it was mixed with equal volume of 0.1 N NaOH. Alkaline layer was decanted and heated at 80°C for 10 min. The orange-yellow colour developed was measured at 450 nm.

#### 2.5.2. Butyrylcholinesterase (BChE) Inhibition Assay

Inhibition of BChE was assessed by a modified colorimetric method of Ellman [14]. The BChE activity was determined in a reaction mixture containing 200 *μ*L of a solution of BChE (0.415 U/mL in 0.1 M phosphate buffer, pH 8.0), 100 *μ*L of a solution of 5,5′-dithiobis(2-nitrobenzoic) acid (3.3 mM in 0.1 M phosphate-buffered solution, pH 7.0) containing NaHCO_3_ (6 mM), juice dilutions (0–100 *μ*L), and 500 *μ*L of phosphate buffer, pH 8.0. After incubation for 20 min at 25°C, butyrylthiocholine iodide (100 *μ*L of 0.05 mM solution) was added as the substrate, and BChE activity was determined with an ultraviolet spectrophotometer from the absorbance changes at 412 nm for 3.0 min at 25°C.

### 2.6. * In Vitro *Antioxidant Studies 

#### 2.6.1. Fe^2+^ Chelation Assay

The Fe^2+^ chelating ability of the infusions was determined using a modified method of Minotti and Aust [15] with a slight modification by Puntel et al. [16]. Freshly prepared 500 *μ*M FeSO_4_ (150 *μ*L) was added to a reaction mixture containing 168 *μ*L 0.1 M Tris-HCl (pH 7.4), 218 *μ*L saline, and the extracts (0–25 *μ*L). The reaction mixture was incubated for 5 min, before the addition of 13 *μ*L 0.25% 1, 10-phenanthroline (w/v). The absorbance was subsequently measured at 510 nm in a spectrophotometer. The Fe (II) chelating ability was subsequently calculated.

#### 2.6.2. 2′-Azino-bis(3-ethylbenzthiazoline-6-sulphonic Acid) (ABTS) Radical Scavenging Ability

The ABTS∗ scavenging ability of the infusions was determined according to the method described by Re et al. [17]. The ABTS∗ was generated by reacting an (7 mmol/l) ABTS aqueous solution with K_2_S_2_O_8_ (2.45 mmol/l, final concentration) in the dark for 16 h and adjusting the Abs734 nm to 0.700 with ethanol. 0.2 mL of appropriate dilution of the extract was added to 2.0 mL ABTS∗ solution and the absorbance were measured at 734 nm after 15 mins. The Trolox equivalent antioxidant capacity was subsequently calculated.

### 2.7. Data Analysis

The results of three replicates were pooled and expressed as mean ± standard deviation (S.D.). Student's *t*-test, one-way analysis of variance (ANOVA), and least significance difference (LSD) were carried out [18]. Significance was accepted at *P* ≤ 0.05.

## 3. Results 

**Figure 1:**
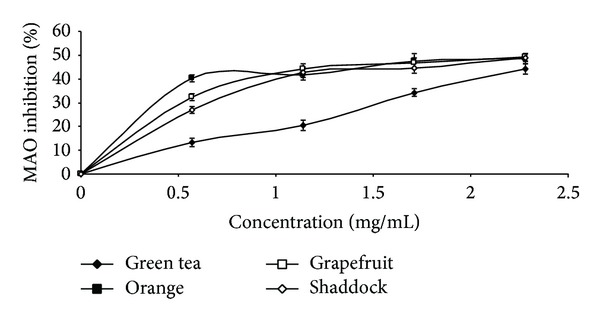
Monoamine oxidase inhibitory activity of infusions of green tea and some citrus peels. Values represent means ± standard deviation of triplicate readings.

**Figure 2:**
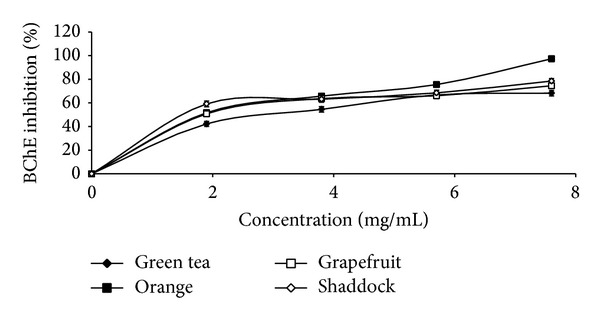
Butyrylcholinesterase inhibitory activity of infusions of green tea and some citrus peels. Values represent means ± standard deviation of triplicate readings.

**Figure 3:**
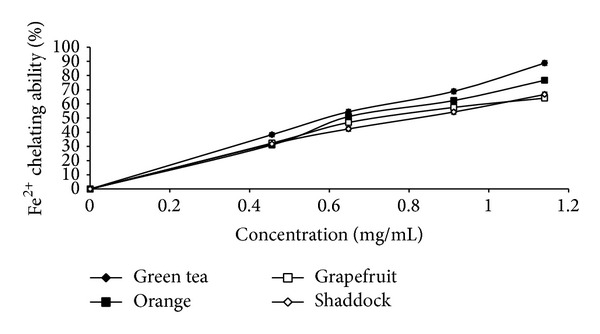
Fe^2+^ chelating abilities of infusions of green tea and some citrus peels. Values represent means ± standard deviation of triplicate readings.

**Figure 4:**
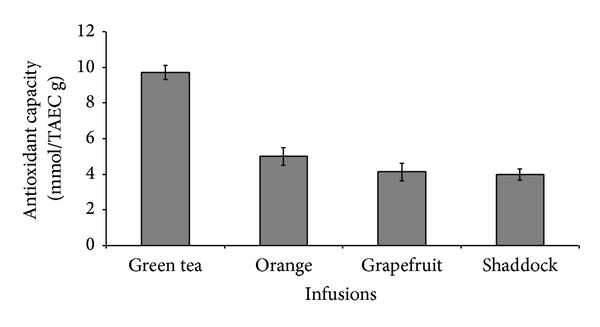
ABTS∗ scavenging ability of infusions of green tea and some citrus peels.

The results of the total phenol and flavonoid contents of the infusions from green tea and the citrus peels as presented in Table 1 revealed that green tea had the highest total phenol (43.3 mg/g) and total flavonoid (16.4 mg/g) content, when compared to orange [total phenol (19.6 mg/g), total flavonoid (6.5 mg/g)], shaddock [total phenol (16.3 mg/g), total flavonoid (5.2 mg/g)], and grapefruit [total phenol (17.7 mg/g), total flavonoid (5.9 mg/g)]. The effects of the infusions on monoamine oxidase and butyrylcholinesterase activities are presented in Figures 1 and 2, respectively, and the EC_50_ values in Table 2. The infusions inhibited the enzymes' activities in a dose-dependent manner. However, orange (EC_50_ = 1.78 mg/mL) had the highest MAO inhibitory ability, while green tea had the least MAO inhibitory ability (EC_50_ = 2.56 mg/mL). Similarly, the BChE inhibitory abilities of the infusions revealed that green tea had the least inhibitory ability (EC_50_ = 5.43 mg/mL) when compared to the other infusions [orange (EC_50_ = 3.61 mg/mL); shaddock (EC_50_ = 4.42 mg/mL); grapefruit (EC_50_ = 4.20 mg/mL)].  Figure 3 and Table 2 revealed that infusions from green tea (EC_50_ = 0.63 mg/mL) had the highest Fe^2+^ chelating ability in comparison to the citrus peels' infusions [orange (EC_50_ = 0.72 mg/mL); grapefruit (EC_50_ = 0.81 mg/mL); shaddock (EC_50_ = 0.82 mg/mL)]. Furthermore, the ABTS radical scavenging abilities of the infusions reported as Trolox equivalent (Figure 4) revealed that green tea (9.71 mmol/g) had the highest ABTS radical scavenging ability, while grapefruit (4.13 mmol/g) and shaddock (3.99 mmol/g) had the least.

## 4. Discussion 

The inhibition of monoamine oxidase and BChE by the infusions used in this study suggests their potential use in the management of neurodegenerative conditions such as AD and PD. This study showed that infusions of grapefruit, orange, and shaddock peels had higher inhibitory effects on these enzymes than green tea infusions. The therapeutic importance of MAO inhibition by the infusions is buttressed by the fact that there is a decrease in the levels of monoamine neurotransmitters (dopamine, serotonin, and norepinephrine) and an increase in the activity of MAO in the brain tissues of AD patients [3, 19]. Therefore, the inhibition of MAO is central in increasing the levels of these neurotransmitters in the brain. More so, MAO inhibitors have been shown from previous studies to reduce the production of reactive oxygen species [3]. The MAO inhibition by the infusions could be linked to the phenolic composition as flavonoid derivatives have been shown to be MAO inhibitors due to their structural similarity to synthetic inhibitors [20]. The cholinesterases are important therapeutic targets in the management of AD [7]. The AChE inhibitory properties of citrus peel aqueous extracts had been earlier reported by the authors [9]. This study showed that infusions from citrus peels had a higher BChE inhibitory effect than green tea infusions. BChE inhibition is very important in AD management as the concentration of BChE rises with the progression of the degenerative condition [5, 6]. Green tea and citrus peels are rich in phenolic compounds, especially flavonoids which have also been shown to possess anticholinesterase properties [21, 22]. It has also been shown that there exists a structural similarity between natural polyphenolic compounds and established cholinesterase inhibitors in terms of molecular weight, phenolic rings, and hydrophobic component [23].

MAO activity has been shown to result in higher free radicals generation [24]. Similarly, previous studies have shown an increase in MAO activity and iron in the neurodegenerative processes leading to Alzheimer's disease and Parkinson's disease [1]. The neuroprotective effects of MAO inhibitors had also been linked to their ability to inhibit the formation of H_2_O_2_ and NH_3_, which are harmful products of amine degradation [25]. Therefore, the radical scavenging abilities and iron chelating properties of the infusions further confirm their potentials as dietary means for the management of neurodegenerative conditions. The antioxidant activities of the green tea and citrus peels' infusions were studied using different in vitro methods due to the complex composition of natural substances. The radicals [1,1-diphenyl-2 picrylhydrazyl (DPPH) and hydroxyl (OH)] scavenging abilities of aqueous extracts of the citrus peels used in this study had been earlier reported by the authors [9]. However, in this study the radical scavenging abilities of infusions from green tea and some citrus peels were studied using a more stable nitrogen-centred radical species (ABTS∗) [17]. The results revealed that green tea had the highest ABTS∗ scavenging ability compared to infusions from the peels.

The Fe^2+^ chelating abilities of the infusions as shown in this study could be of therapeutic benefit in AD management as iron chelators have demonstrated beneficial effects in AD patients [26] by slowing the progression of the disease. Iron has been shown to accumulate in the brain of AD patients [27] and generates radicals through the Fenton reaction [28]. Synthetic iron chelators have the disadvantage of high toxicity and difficulty crossing the blood brain barrier [29]. However, the infusions used in this study are rich in phenolic compounds which are iron chelators and can cross the blood brain barrier [1, 30]. Other studies have also suggested that MAO inhibitors and iron chelators would be effective in the management of neurodegenerative conditions [31]. Therefore, novel compounds which are combined iron chelators and MAO inhibitors have been developed. The bifunctional ability of the infusions used in this study to inhibit MAO and chelate Fe^2+^ could be part of the mechanism by which green tea and citrus peels could manage or prevent neurodegeneration.

The high total phenolic and flavonoid contents of the green tea and citrus peels' infusions agree with earlier research articles [32, 33]. Furthermore, the total phenolics and flavonoids contents of the citrus peels varied between the citrus species tested (shaddock, grapefruit, and orange). The high total phenolics content of the peels agreed with earlier reports on the phenolics content of some citrus peels. These variations could be due to factors such as the genetic potential of individual species for biosynthesis of secondary metabolites such as polyphenols [34].

## 5. Conclusion 

The antioxidant properties and inhibition of MAO and BChE by the green tea and citrus peels infusions make them good dietary means for the management of neurodegenerative conditions. However, further* in vivo* experiments and clinical trials are recommended.

## Figures and Tables

**Table 1:** Total phenolic and flavonoid contents of infusions of green tea and some citrus peels.

Sample	Total phenol (mg GAE/g)	Total flavonoid (mg QE/g)
Green tea	43.3 ± 2.3^a^	16.0 ± 2.1^a^
Orange	19.6 ± 1.1^b^	6.5 ± 1.2^b^
Shaddock	16.3 ± 1.1^b^	5.2 ± 0.9^b^
Grapefruit	17.7 ± 1.2^b^	5.9 ± 0.8^b^

Values represent means of triplicate. Values with the same alphabet along the same column are not significantly different (*P* > 0.05).

GAE, gallic acid equivalents; QE, quercetin equivalents.

**Table 2:** EC_50_ values of inhibition of monoamine oxidase, butyrylcholinesterase, and Fe^2+^ chelating abilities of green tea and some citrus peels' infusions.

	Green tea	Orange	Grapefruit	Shaddock
MAO inhibition	2.56 ± 0.09^a^	1.78 ± 0.11^b^	2.00 ± 0.08^c^	1.93 ± 0.06^c^
BChE inhibition	5.43 ± 0.10^a^	3.61 ± 0.06^b^	4.20 ± 0.09^c^	4.42 ± 0.07^d^
Fe^2+^ chelation	0.63 ± 0.11^a^	0.72 ± 0.09^b^	0.81 ± 0.07^c^	0.82 ± 0.09^c^

Values represent means of triplicate. Values with the same alphabet along the same row are not significantly different (*P* > 0.05).
